# Dynamic geographical accessibility assessments to improve health equity: protocol for a test case in Cali, Colombia

**DOI:** 10.12688/f1000research.127294.1

**Published:** 2022-11-28

**Authors:** Luis Gabriel Cuervo, Ciro Jaramillo, Daniel Cuervo, Eliana Martínez-Herrera, Janet Hatcher-Roberts, Luis Fernando Pinilla, María Olga Bula, Lyda Osorio, Pablo Zapata, Felipe Piquero Villegas, Maria Beatriz Ospina, Carmen Juliana Villamizar

**Affiliations:** 1 Department of Paediatrics, Obstetrics & Gynaecology and Preventative Medicine, Universitat Autònoma de Barcelona, Barcelona, Catalonia, Spain; 2 School of Civil and Geomatic Engineering, Universidad del Valle, Cali, Valle del Cauca, Colombia; 3 IQuartil SAS, Bogotá D.C., 110111-300, Colombia; 4 Facultad Nacional de Salud Pública, Universidad de Antioquia, Medellín, Antioquia, Colombia; 5 WHO Collaborating Centre for Knowledge Translation and Health Technology Assessment for Health Equity, Bruyère Research Institute, University of Ottawa, Ottawa, Ontario, K1R6M1, Canada; 6 Universidad de la Sabana, Bogotá D.C., Colombia; 7 Egis Consulting, Bogotá D.C., Colombia; 8 School of Public Health, Universidad del Valle, Cali, Valle del Cauca, Colombia; 9 Author of patobiography, Independent Patient Representative, Bogotá, D.C., Colombia; 10 Department of Public Health Sciences, Faculty of Health Sciences, Queen's University, Kingston, ON, K7L 3N6, Canada; 11 Johns Hopkins Bloomberg School of Public Health, Baltimore, Maryland, USA

**Keywords:** Health services accessibility, City planning, Urban health, Health inequality monitoring, Spatial Analysis, Residence characteristics

## Abstract

This protocol proposes an approach to assessing the place of residence as a spatial determinant of health in cities where traffic congestion might impact health services accessibility. The study provides dynamic travel times presenting data in ways that help shape decisions and spur action by diverse stakeholders and sectors.

Equity assessments in geographical accessibility to health services typically rely on static metrics, such as distance or average travel times. This new approach uses dynamic spatial accessibility measures providing travel times from the place of residence to the health service with the shortest journey time. It will show the interplay between traffic congestion, accessibility, and health equity and should be used to inform urban and health services monitoring and planning.

Available digitised data enable efficient and accurate accessibility measurements for urban areas using publicly available sources and provide disaggregated sociodemographic information and an equity perspective.

Test cases are done for urgent and frequent care (i.e., repeated ambulatory care). Situational analyses will be done with cross-sectional urban assessments; estimated potential improvements will be made for one or two new services, and findings will inform recommendations and future studies.

This study will use visualisations and descriptive statistics to allow non-specialized stakeholders to understand the effects of accessibility on populations and health equity. This includes “time-to-destination” metrics or the proportion of the people that can reach a service by car within a given travel time threshold from the place of residence.

The study is part of the AMORE Collaborative Project, in which a diverse group of stakeholders seeks to address equity for accessibility to essential health services, including health service users and providers, authorities, and community members, including academia.

## Introduction

Equitable accessibility is central to the United Nations Sustainable Development Goals and targets like universal health coverage and quality of care. ^1^^–^^5^ A common definition of accessibility is the relative ease (travel time by car) by which a destination (health service) can be reached from a given location (residence). ^6^^–^^8^ Equity assessments in geographical accessibility to health services typically rely on static metrics, such as distance or average travel times. ^9^^–^^11^

Measuring equitable accessibility has several challenges. Accessibility studies assess distance or the shortest average travel time to the nearest facility; they seldom assess equity and are typically geared towards field experts. ^12^^–^^14^ These studies usually explore broad service categories without focusing on specific services people might need. They are lengthy, costly, and rarely address the dynamic temporospatial nature of accessibility, such as its links to traffic congestion. ^6^^,^^15^^,^^16^

Reliable data on equity of accessibility to urban health services has been elusive for most cities due to limitations in sampling techniques, extrapolations, and the use of complex methods to capture temporospatial variations associated with traffic congestion. Stakeholders, including urban and health service planners, have relied on indirect fixed assessments that fail to address the impact of traffic congestion on equity. ^6^^,^^9^^,^^15^^,^^17^

These challenges were understandable because assessments were cumbersome and required detailed origin-destination studies with small samples from home surveys, traffic corridor speed cameras, or extrapolations from average traffic in selected corridors, with limited intersectoral and multistakeholder participation. ^6^^,^^16^^,^^18^^–^^26^ Results would turn irrelevant given the rapid changes in conditions, including traffic congestion, populations, or infrastructure. ^27^

Travel times affect geographic accessibility and the quality of care. ^1^^,^^28^^,^^29^ Poor accessibility can lead the most socially disadvantaged populations to pay the highest share to reach health services, an aberration of social justice known as the “inverse care law.” Lengthy travel times hurt people; they are detrimental to health, well-being, and family finances. ^28^^,^^30^^–^^35^ Measuring travel times might reveal problems hiding in plain sight. Addressing accessibility might help people unable to choose a better place of residence overcome structural barriers to health. ^36^

Travel time assessments have been widely available for commodities and commerce and powered consumer apps. These developments have yet to translate into a systematic integration of dynamic equity assessments into urban and health services planning or public sector debates about land use and how to put health services within reach of the broadest population possible.

Measurements have proven difficult, and this project explores a new approach to making measurements feasible, scalable, and adaptable to urban sprawl and lengthening journeys. This new line of research explores if market forces and land use plans achieve service accessibility and if this holds for populations in situations of vulnerability. ^9^^,^^12^^,^^29^

## The need for this study

This proposal spurs the scaling up and replicating of accessibility analyses to health services in urban centers while promoting accessibility indicators based on dynamic travel times. The research demystifies the use of big data and analytics that reveal the needs of citizens, including the most vulnerable. ^37^^–^^42^ The project will lay the basis for subsequent studies that assess the value and use of dynamic accessibility and equity assessments in urban and health services planning.

This project explores a new approach to making such measurements feasible, scalable, and adaptable to urban sprawl and long journeys. This new line of research examines whether market forces and land use planning achieve services’ accessibility and if this holds for populations in situations of vulnerability. ^29^^,^^43^

Using reliable data that is systematically updated and publicly available could be a game-changer. ^6^^,^^37^^,^^44^^,^^45^ This study tests a new approach for assessing dynamic accessibility to health services, providing an equity perspective and using digital data sources. ^9^^,^^44^^,^^46^

Using data readily available in the public domain allows these assessments to be completed in a shorter time and with a lower budget. When combined, the growing millions of measurements of travel times (big data) passively collected by mobile apps, the digitalized georeferenced sociodemographic data from the census, and the geolocation of health services, provide a dynamic assessment of accessibility that accounts for temporospatial variations related to traffic congestion. ^6^^,^^46^^,^^47^ Big data provides millions of measurements that allow identifying unexpected correlations with a level of detail and accuracy that surveys and inferences cannot match. ^45^

This study aims to overcome the limitations of regular accessibility assessments by prioritizing dynamic travel times and adopting recommended knowledge production and use practices. The following section details some key features and good practices that contribute to addressing present challenges:

• **Multistakeholder engagement:** a diverse intersectoral team of stakeholders contributes to the AMORE Project throughout the research process, and their inputs also informed the AMORE Platform conceptualization and development. ^48^^–^^51^ Contributors to the AMORE Project Collaborative Group represent the government, community, health service providers, and end users (consumers) who may directly or indirectly shape decisions, policies, plans, and programs. ^48^^,^^52^^–^^54^^,^^55^
• **Measurement** of dynamic travel times using “time to destination” is a universal and comparable metric used by urban dwellers and users of navigation and travel apps. ^44^
• **Digitization and datafication** by using anonymized publicly available georeferenced data of housing, people, and services, including disaggregated sociodemographic characteristics. ^6^^,^^27^^,^^37^
• **Using analytics and modelling** to obtain reasonable estimates and maintain efficiencies and affordability while still delivering valid and reliable forecasts.
• **Disaggregating sociodemographic data** to deliver an equity analysis of accessibility. ^56^^–^^58^
• **Scalability and replicability** using sources increasingly available to low- medium, and high-income settings. The approach can be scaled, adapted, and replicated to other locations, transportation means, services, or sectors.

Subsequent research will explore if revealing territorial inequities in urban and health services planning could catalyze intersectoral responses. ^30^^,^^59^^–^^63^ Intersectoral collaborations rarely occur naturally and are challenged by the lack of consensus on issues and metrics. Using metrics and methods that all parties understand and facilitate direct communication could contribute to intersectoral action; assessing this will require additional research and is the subject of a separate proposal. ^9^^,^^60^^,^^64^^–^^68^

## Objectives

### General objective

To assess dynamic accessibility assessments for selected healthcare services in urban Cali, Colombia, and predict the maximum improvements possible if new services were added.

### Specific objectives

• To assess the temporospatial characteristics of equity and accessibility to hemodialysis, radiation therapy (radiotherapy), and tertiary care emergency services when traveling by car in urban Cali, Colombia.
• To provide dynamic assessments based on selected (arbitrary) travel time thresholds.
• To assess if populations in a situation of vulnerability needing hemodialysis, radiotherapy, and tertiary care emergency services will likely incur longer journeys when traveling by car in urban Cali, Colombia.
• To identify common variations of dynamic accessibility at two moments of the COVID-19 pandemic from an equity perspective.
• Assess the magnitude of absolute and relative accessibility variations attributed to traffic congestion.
• To estimate potential improvement for accessibility gained by expanding services.

Box 1 provides a plain language summary of the project. The *Extended data* contains the goals of the AMORE project and previous versions of the protocol. ^69^^,^^70^^,^^71^

Box 1. **Overview of the study.**

**What is already known on this topic** – dynamic travel times are not available for most cities, including Cali, and are not integrated into urban and health service planning; dynamic geospatial analyses reveal the effects of traffic congestion on health equity and accessibility to health services, a determinant of health.

**What this study adds** – it will provide estimates of accessibility with an equity perspective using simple methods and metrics that concerned stakeholders might find familiar. This will test if dynamic accessibility assessments can be done with existing data. The study adds an approach to analyzing dynamic geographic accessibility by tapping into hundreds of thousands or millions of observations to analyze, predict, improve geographic accessibility to health services, and identify new correlations.

**How this study might affect research, practice, or policy** – This study will provide a new metric and data source to address inequities aggravated by poor accessibility offering new perspectives on land use and the expansion of health services. The study prioritizes travel times over distance, accounting for the temporospatial variations caused by traffic congestion. Subsequent examinations can explore stakeholders' valuing of the data and their communication of the methods and findings with peers and counterparts.

## Protocol

### Ethics

This health services quality improvement protocol will use anonymized coded secondary data sources from publicly available open records and will not include human subjects’ research.

This cross-sectional study will use a research design of GIS modelling applied to case studies based on analyses of publicly available secondary data. This study will conduct paired cross-sectional assessments comparing equity in health services accessibility from the 6 ^th^ to the 12 ^th^ of July 2020 and from the 23 ^rd^ to the 29 ^th^ of November 2020.

Reporting of study results will follow the STROBE Guideline for cross-sectional observational studies and incorporate elements from other guidelines, such as those on equity assessments (CONSORT-E and PRISMA-E equity extensions), public health and policy interventions (TIDieR-PHP), reporting of analytical models (e.g., SPIRIT-AI extension), and multistakeholder engagement with patient and public participation in research (GRIPP2). ^57^^,^^72^^–^^74^

### Context and study population

The study will be a proof-of-concept for implementation in Cali (estimated 2,258 million in 2020), the third largest and most populous city in Colombia and the dominant urban center of Colombia’s southwest and pacific regions (approx. 564 km ^2^). The study includes the entire urban population. Nearly half of Cali’s population lives in low-income housing, 41% in medium income, and 9% in high-income housing. About 84% of the population identifies as white descent, and 14% identify as afro-descendent, with a small proportion identifying as Indigenous or Rrom. ^78^^–^^80^

The COVID-19 pandemic severely impacted the local economy. By January 2021, unemployment rates in Cali rose to 23.2% for women and 14.6% for men, a one-year increase of 8.1% and 3.1%, respectively. The situation was worse for the youth, with an estimated 52% of women and 47.2% of men dependent on the informal economy. One in five people was unemployed, and unemployment rates were substantially higher for those living in lower socioeconomic areas. Cali absorbed 139,000 migrants from Venezuela over the past five years, with more than 25,000 in 2020. ^79^^,^^80^

For the reports, contextual data will be obtained to provide an overview of the use and demand of services. Sources include reports and platforms such as the “Cuentas de Alto Costo.” ^82^^–^^84^

In preparatory dialogues with contributors, we learned about plans to transform Cali into a Special District with its twenty-two communes converted into six to eight minor districts to be led by minor district mayors. ^85^ This new political and administrative layout might raise interest in this topic as new authorities might want to discuss accessibility and equity issues with their constituents and in power-brokering negotiations, noting the equity implications of the concentration of health services in a few sectors of the city.

### Data sources

The study will use anonymized, aggregated data from the following data sources:

• Microdata of Colombia’s National Census for Cali 2018 will be downloaded from the official public website of the National Department of Statistics– DANE. ^86^ This will provide sociodemographic data of the populations at the block level for the entire city. The census population had a 28.1% adjustment estimated for 2020 to account for intercensal growth, under-registration, and migration. ^78^^,^^79^^,^^81^^,^^87^
• The city’s transportation analysis areas (TAZ) and census administrative sectorization for urban Cali will be obtained from the IDESC portal. ^88^ This data allows linking TAZs with city blocks. TAZs are adequate to estimate travel times and less detailed than blocks, thus reducing the number of travel time measurements and adding anonymity to the population.
• Approved health services relevant to the chosen scenarios, will be obtained from the National Special Registry of health services providers – REPS from the Ministry of Health and Social Protection. The services geolocation will be verified with Google Maps. Approved services were checked in June and October 2020 and January 2021, finding they remained unchanged. This protocol will assess accessibility to the entire city’s fourteen tertiary care hospitals with emergency services (REPS Code “Alta complejidad” + 501); eleven hemodialysis units totaling 370 chairs (REPS code 733); five radiotherapy services (REPS code 711).
• Google’s Distance Matrix API provides big data measurements of travel times from the origin (TAZ for the residence) to the destination (TAZ of the health service). It allows the identification of travel time changes during the assessed weeks.

### Data integration: The AMORE Platform

Secondary data will be integrated into the AMORE Platform, a web-based digital platform developed with inputs and feedback from stakeholders and piloted by the AMORE Project. ^89^^–^^91^ The Platform is hosted by IQuartil SAS. See https://www.iquartil.net/proyectoAMORE .

The AMORE Platform was developed and tested following a design-thinking approach between June and August 2020. ^49^^,^^92^^–^^95^ The final version was completed in February 2022. The digital web-based platform was developed for this project by the principal investigator with input from experts in data science, public health, logistics, and mobility and a wide range of stakeholders (A description of the development and piloting phases of the AMORE Platform can be provided).

**Figure 1.**
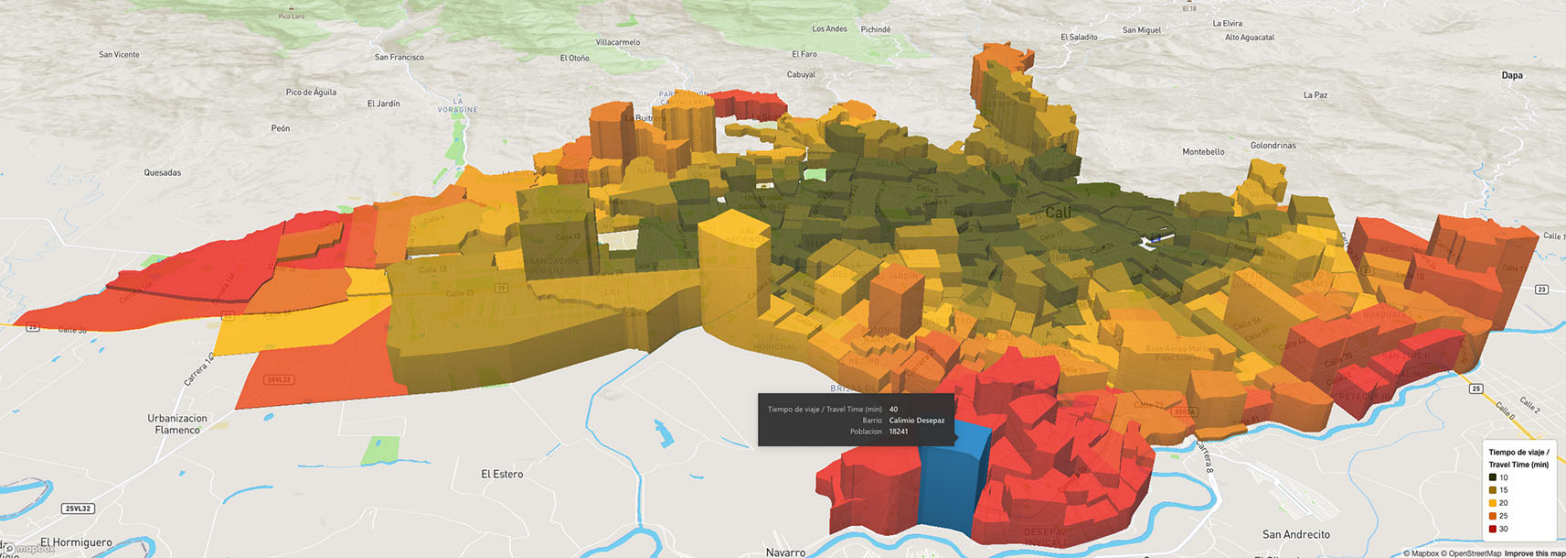
Cali, accessibility to tertiary care emergency service late morning to early afternoon Mon-Sat. North to the right and west at the top. Source: AMORE Platform. ©Mapbox © OpenStreetMap

**Figure 2.**
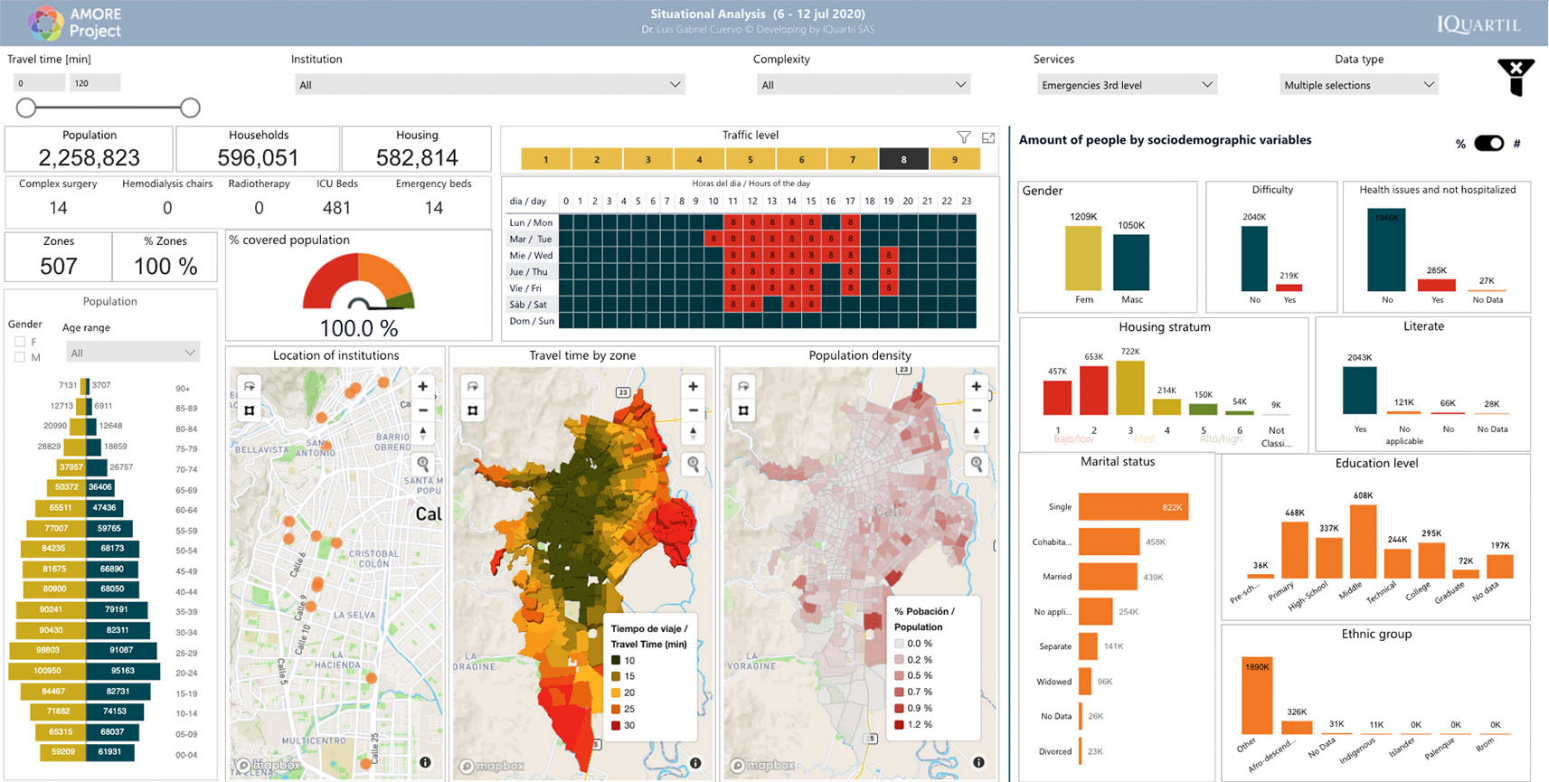
AMORE Platform Interface for situational analysis for tertiary care emergencies. Source: AMORE Platform. ©Mapbox

**Figure 3.**
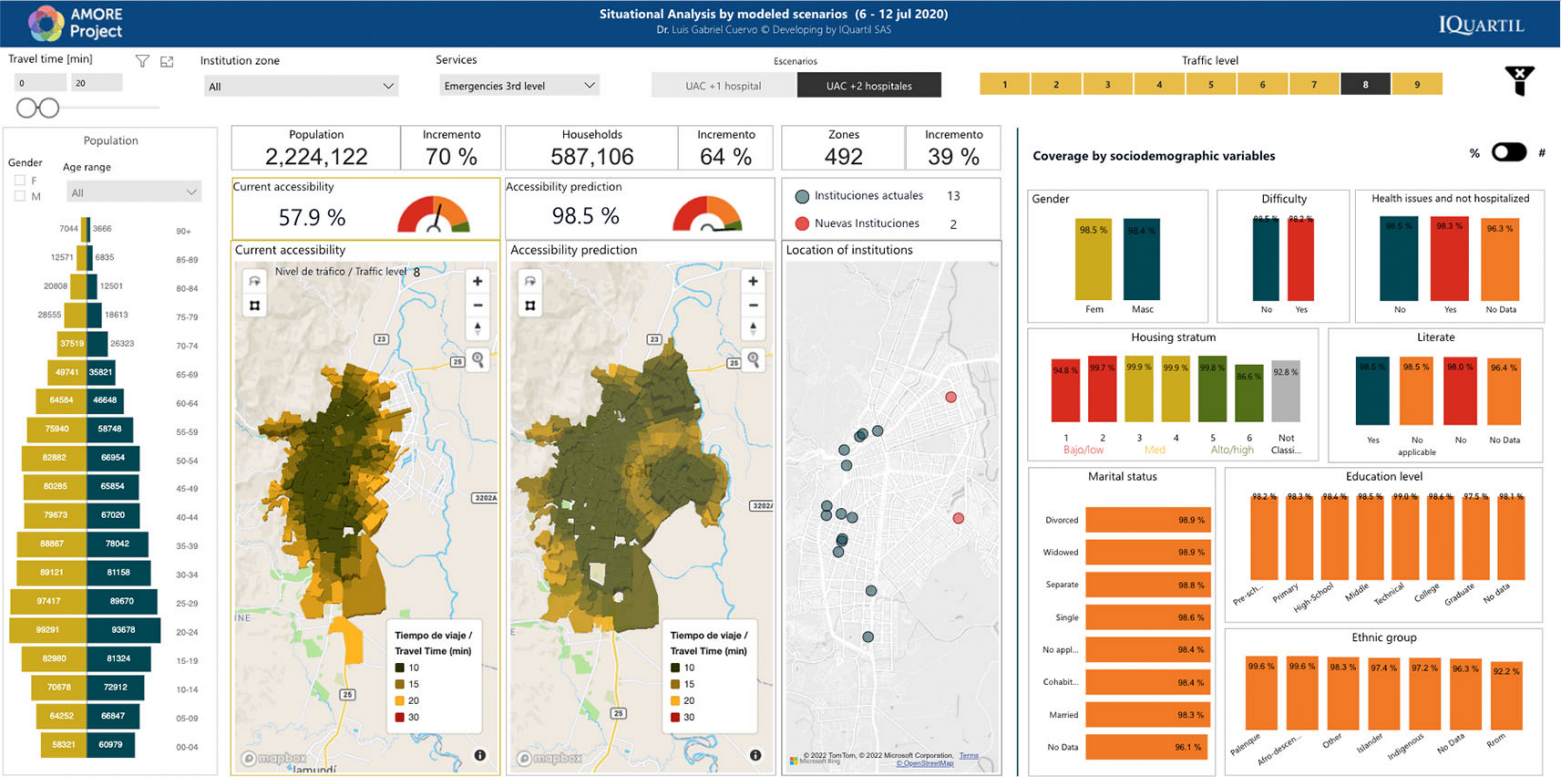
AMORE Platform with predictive analyses of adding two new tertiary care emergency services. Source: AMORE Platform. ©Mapbox

Figure 1, Figure 2, and Figure 3 display examples of the AMORE Platform’s interface or “ front - end ” panels (presentation layers) with its zoomable choropleth maps and graphics that integrate multiple layers of data. Filters activated by tapping on the graphs act on sociodemographic variables, travel times, and health services to offer a descriptive analysis. The front-end has been developed with Microsoft's Power BI™ (v. 2.102.683.0). The back end (data access layer) is written in Python (v. 3.9.10)™ open-source software from the Python Software Foundation – PSF and in the Konstanz Information Miner – KNIME (v 4.5.1), a free and open-source data analytics, reporting, and integration platform *.*

### Study variables

Reports will describe the people and percentages of the entire population able to reach services within a set threshold with peak and free-flow traffic conditions for each scenario. The variations in these accessibility figures will be disaggregated by sociodemographic characteristics such as sex, ethnicity, the socioeconomic stratum of housing, maximum education attainment, and marital status. These reports will contrast the statistics for peak and free-flow traffic conditions. These reports will use an arbitrary 15-minute threshold for accessibility by car to tertiary care emergencies and 20-minutes for hemodialysis and radiotherapy. Colombia’s census provides a binary sex classification (male-female) based on self-reporting. ^86^^,^^96^

**Figure 4.**
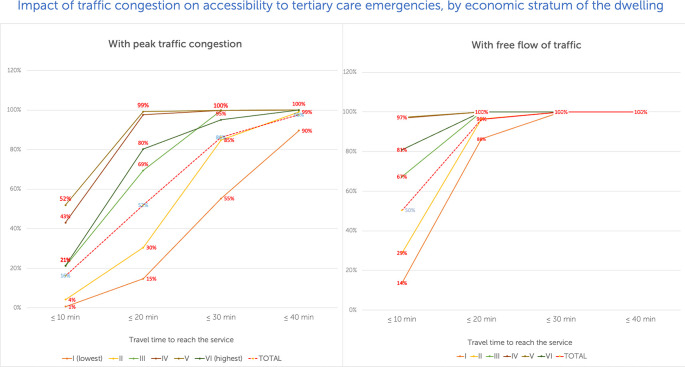
Comparing accessibility by socioeconomic stratum, tertiary care emergencies. ^97^

Graphs will be used to present variations in accessibility as traffic congestion increases for different travel time intervals (e.g., 10-minute intervals vs. accessibility for each socioeconomic stratum), as shown in Figure 4. The contrast will be drawn between results obtained for July and November 2020. Reports will include the location(s) maximizing accessibility if one or two new services are added, contrasting predicted accessibility vs. measured accessibility for July and November 2020, and the recommended services locations. For an example, see Figure 3. All estimates in this test case use travel by car. Reports will include visualizations from the AMORE Platform, tables, and simple graphs with descriptive statistics.

### Data analysis

Data and images for analyses will be obtained from the AMORE Platform and presented using descriptive statistics for absolute figures (people) and relative (percentage of population), as shown in Figure 2 and Figure 3.

The use of relatable and commonly used metrics (i.e., time to destination), descriptive statistics (percentage of a population that can reach the services within a travel time threshold), visualizations, and simple graphs (e.g., Figure 2, Figure 3, Figure 4) and maps (e.g., Figure 1) are chosen to allow non-specialized stakeholders to understand the effects of accessibility on populations and health equity.

The study will deliver (1) situational analyses of accessibility to a selection of urgent or frequent health care services scenarios in urban Cali, Colombia, and (2) predictions of potential improvements to the accessibility of adding services under changing assumptions.

Arbitrary travel-time points will be used (15 minutes for urgent care; 20 minutes for frequent care services); we found no standards for travel time thresholds.

The situational analyses of accessibility will consider the following study scenarios:

1. **Urgent care,** assessing travel times to the health emergency department with the shortest journey by car, among the 14 hospitals with tertiary care emergency departments.
2. **Frequent care,** assessing travel times to ambulatory services that require regular use. The analyses will investigate the shortest journey to five radiation therapy services and eleven hemodialysis units.
  For these case studies, the project will deliver a:
  • **Situational analysis** uses descriptive and diagnostic analytics to assess accessibility under traffic conditions. Choropleth maps mark the boundaries of travel times Figure 1. The blocks building the map represent Traffic Analysis Zones (TAZ), and their height in the 3D choropleth map represents population density. By pointing to a TAZ, identification, population, and travel time to the nearest facility are displayed, and the sociodemographic characteristics of the people are presented in a dashboard ( Figure 2). The dashboard also includes a choropleth map with the density of the population being analyzed.

**Optimization analysis** using predictive and prescriptive analytics for modelling. Using heuristic techniques, the research will predict accessibility with different service schedules or when adding up to two new services in locations that maximize accessibility. Case studies will focus on hemodialysis and radiotherapy, which are used to treat high-cost conditions and must be accessed repeatedly for prolonged periods. For example, hemodialysis usually requires 3-5 weekly sessions, and radiotherapy may require daily sessions for weeks. Predicted accessibility with new services will be compared with measured accessibility ( Figure 3).

### Preparation for the project

Beginning on 12 ^th^ June 12 2020, interviews were conducted with key informants, local authorities, and experts to inform the project, assess feasibility, and develop a protocol. They provided verbal consent to participate. These interviews offered contextual insights and ideas for the AMORE Platform to address the needs of data users and stakeholders. Discussions helped identify contributors and provided valuable contextual information. They also revealed how stakeholders approached health equity and accessibility and the relationship to urban and health services planning and land use. Stakeholders offered insights into the elements and processes of urban and health services planning, public policy, and advocacy.

Interviewees included:

• Members of the SIGELO Project on vulnerability, accessibility, and logistics in the context of the COVID-19 pandemic, Universidad del Valle
• Health equity and public health experts at the Bruyère Institute, University of Ottawa
• Contributors to Cali’s Administrative Department of Municipal Planning (DAPM)
• Advisors and staff working with Cali’s local government, including the secretariats of Health, Mobility, Urban planning, and Emergency response and preparedness.
• Data Science for All Team33 and IQuartil SAS analysts.
• Former local government and education authorities
• Urban observatories, urbanists, and networks on urbanism, mobility, and public health.
• Innovators and advisors working with health services and systems
• Service providers, including managers and science advisors
• Health services and accessibility data users
• Doctoral and professional networks
• Designers, graphic and science communications professionals, and artists
• Researchers and research sponsors.

Overall, 28 meetings were held with key informants and stakeholders between July 2020 and March 2022, when the advanced prototype of the AMORE Platform was completed. Like every doctoral thesis, this project is subject of yearly follow-up reviews by the Commission of the Doctoral Program on Research Methodology for Biomedical Research and Public Health of the Universitat Autònoma de Barcelona, since October 2020.

During the preparatory phase, the project was also debated in international fora such as the Global Health Learning Network of the University of Ottawa, The 4 ^th^ Urban Forum “Lima Cómo Vamos,” the CEDEUS-REDEUSLAC II International Symposium of Doctoral Candidates on Urban Development and Sustainability for Latin America, and the Caribbean (Chile), and with urban observatories (Cali, Bucaramanga). ^89^^,^^98^^,^^99^

These interviews shaped the AMORE project and platform. The objectives and platform were discussed from a theoretical perspective. Once the prototypes of the AMORE Platform became available, tests and demonstrations were made as part of the validation.

Data downloads and sources were stored in a dedicated repository using CSV formats to adhere to data sharing and reuse good practices. They will be made public with the publication of relevant research reports. Similarly, the AMORE Platform hosted by IQuartil SAS is made accessible with the completion of non-disclosure agreements. We expect to make platform sections publicly available as relevant results are published. ^100^

### Fidelity/adaptation

The fidelity of the AMORE Platform is based on data validation and verification exercises, comparing the findings from the AMORE Platform using the two data downloads and the two development teams.

The results of the platform will likely be optimistic for several reasons. For example, people do not always travel to the service with the shortest journey for known (e.g., lack of coverage from the insurance in the institution) and unexpected reasons (familiarity, poor navigation aids, reputation). The potential for improved accessibility would be accurate if all people were entitled to access those services.

The census includes respondent-reported data that is subject to interpretation. For example, variables like disability and health status are self-reported, and the question is unspecific.

Respondents may not find a suitable response option. For example, ethnicity has no category for Caucasian or mestizo populations representing a substantial part of the population. People with mixed backgrounds may find no suitable option to represent them.

The census is still well suited for this study: the data has been digitized, and sociodemographic data are linked with the residential block. The place of residence is a common starting point for people undergoing hemodialysis or radiotherapy, children, the elderly, and those not engaged in formal employment.

Mobile phone data is impractical because it cannot be accurately tied to reliable sociodemographic data; coverage varies among the population and excludes those unregistered as users or without a phone. It also has technical limitations. ^101^

Accessibility and spatial equity have been studied in Cali by the Research Group on Transport, Transit and Roads (GITTV) of the Faculty of Engineering of the Universidad del Valle. Members of this group contributed to the validation of the AMORE Platform, and the preliminary findings of the AMORE Platform are consistent with those of the GITTV and other authors. ^102^^–^^107^

### Harms, risks, and ethical considerations

This observational study addresses the impact of mobility on health equity without researching human subjects and by integrating anonymized coded secondary data obtained from openly available records.

The study does not involve human subjects’ research. The AMORE Platform and dynamic geospatial analyses expose social justice issues and potential solutions of benefit to society by enabling informed decisions relevant to policies, plans, and procedures for improving health equity. This data can also predict or monitor changes in urban accessibility. The data used is anonymized and publicly available. Under Colombian law, this component fits the definition of research without risk, as described in Resolution 008430-1993 of the Ministry of Health. ^108^ This was corroborated on 25 ^th^ July 2022, by the Research Ethics Committee of the School of Engineering of the Universidad del Valle, which declared the project “without risk” per Colombian law (Ref: CEIFI 010-2022). The project was cleared on September 16, 2022 by the Commission on Ethics in Animal and Human Experimentation (CEEAH), and the Vice-Rector for Research, Universitat Autònoma de Barcelona (Ref: CEEAH-6100) on September 20, 2022.

The study can challenge current thinking with data and disrupt traditional approaches to land use and health services planning that may perpetuate pervasive inequalities that could fuel social strife and corruption. ^102^^–^^105^^,^^109^^–^^115^

The ethical approach of this study follows the broader principles and considerations of public health ethics and health systems ethics; it generates population data valuable to address inequity and social injustice, is helpful for accountability and is relevant to intersectoral action. ^116^^,^^117^ The need for further guidance on these issues remains a challenge for cross-sectoral collaboration. It is part of the ongoing discussions on stakeholder engagement in global health. ^73^^,^^117^

Accessibility is a determinant of health on the supply side of health equity. ^119^ Useful, valid equity assessments in accessibility matter to health systems and social justice. Having action-oriented data to challenge established thinking might contribute to various SDGs, such as improving good health and well-being (SDG 3), reducing inequalities (SDG10), having sustainable cities and communities (SDG11), improving infrastructure (SDG 9), and facilitating partnerships to achieve the SDGs (SDG17). ^3^^,^^48^^,^^52^^,^^60^^,^^116^

These ideals are synergic with other urban development and planning initiatives on a human scale: Smart City, the 15-minute City, the Caring City, and the Committed City. Having data is the first step for technology to serve the needs of urban dwellers and inform public policy regularly or when facing health emergencies and pervasive inequities. ^52^^,^^120^^–^^122^

The risks of this study are especially those associated with data science and artificial intelligence. Travel time data providers do not disclose the algorithms they use. These are empirically known to be accurate and are expected to be more accurate for the areas most travelled by people with network-engaged smartphones and sites where infrastructure and conditions remain stable; accuracy may vary across the city. ^27^^,^^123^^,^^124^

There is a risk of errors in programming or labelling data; to control this risk, the validity of the data was tested, repeatedly reviewed, and found sensible by experts and local contributors. The chance that inaccuracies result from clustering traffic and times is low and is unlikely to change the overall picture the project analyzes.

The project reveals accessibility levels for populations and sectors of the city. It uses heuristic analysis to identify areas in which new services would significantly impact accessibility. These areas, like traffic conditions, may evolve. However, the data provided to inform decisions gives an overall idea of the locations that would optimize accessibility. It is unlikely that conditions and populations would change fast enough to make those broad estimations suddenly irrelevant. Regular updating of the AMORE Platform would allow for assessing these variations and would be the subject of further studies after this test case. Additional factors influence the use of a service, including insurance coverage entitlements. Exploring this would require different data layers and funding that exceeds the purpose and scope of the test case and would be a matter of subsequent implementation. There is an inherent risk of revealing social injustices or inequities that can lead to discomfort, alienation, or corrective action.

### Dissemination, promotion, and implementation of findings

As part of the project, communication tools such as animations , infographics, videos, summaries, and logos were developed.

The research team seeks to publish its reports in open-access impactful journals and present them to diverse audiences, including observatories, networks, and intersectoral groups.

The planned reports include:

• Accessibility of health services for the urban population of Cali, 2020: urgent care and frequent care
• Predicted accessibility of health services for the urban population of Cali with the addition of health services in areas that would maximize accessibility (urgent care and frequent care scenarios) or changes in service schedules (frequent care)
• Editorials and methodological articles.

### Protocol study status

Data extraction for research reports was initiated in January 2022 and is underway.

## Data Availability

The Data Sources section provides a list of publicly available data sources that will be used in this project. Relevant interfaces for each case study will be published with research results at https://www.iquartil.net/proyectoAMORE/. Figshare: Theory of Change for AMORE Project Protocol 2022 https://doi.org/10.6084/m9.figshare.20485404.v2. ^69^ This project contains the following extended data:
- The 2022 goals of the AMORE project and how they will be achieved Open Science Framework: Dynamic geographical accessibility assessments to improve health equity: protocol for a test case in Cali, Colombia. https://doi.org/10.17605/OSF.IO/ETPMA. ^70^ This project contains the following extended data:
- 20220726 Protocol 5.5 AMORE Project_Approved CEII-UdelV Assembled.pdf - 20220726 Protocol 5.6.1 AMORE Project_Approved CEIIFI - CEEAH.pdf Data are available under the terms of the Creative Commons Attribution 4.0 International license (CC-BY 4.0).
