# Supplementary material for: Dynamic geographical accessibility assessments to improve health equity: protocol for a test case in Cali, Colombia
Source: F1000Res. 2022 Nov 28;11:1394. [Version 1] doi: 10.12688/f1000research.127294.1 (PMC10352632; doi:10.12688/f1000research.127294.1)
Supplement: Peer review history

# Peer review: Reviewer response for version 1

**DOI:** 10.5256/f1000research.139788.r175826

**Published:** 2023-07-17

**Authors:** Luiz Galvao

**Affiliations:** 1 Oswaldo Cruz Foundation, Rio de Janeiro, Brazil

The paper proposes a new approach to assessing the accessibility of health services in urban areas. The approach uses dynamic travel times, which take into account traffic congestion, to assess the ease with which people can reach health services from their homes. The paper also takes an equity perspective, assessing how the accessibility of health services varies for different populations. It argues that the traditional approach to assessing accessibility, which uses static metrics such as distance or average travel time, is no longer adequate in the context of urban sprawl and traffic congestion. Dynamic travel times provide a more accurate measure of accessibility, as they take into account the real-world conditions that people face when trying to reach health services.

 The paper uses data from Cali, Colombia, to show that people in vulnerable populations, such as those living in poverty or those with disabilities, often have less access to health services than people in more privileged populations.

 The conclusions includes a new framework for assessing dynamic accessibility to health services. The framework takes into account the following factors:

- The location of health services
- The location of people's homes
- Traffic congestion
- The sociodemographic characteristics of the population

The paper argues that the framework can be used to identify areas where accessibility to health services is poor, and to target interventions to improve accessibility.

 Overall, the paper is a valuable contribution to the literature on accessibility to health services. The new framework proposed by the authors has the potential to improve the way that accessibility to health services is assessed and improved.

Is the study design appropriate for the research question?

Yes

Is the rationale for, and objectives of, the study clearly described?

Yes

Are sufficient details of the methods provided to allow replication by others?

Yes

Are the datasets clearly presented in a useable and accessible format?

Yes

Reviewer Expertise:

environmental and global health

I confirm that I have read this submission and believe that I have an appropriate level of expertise to confirm that it is of an acceptable scientific standard.

# Peer review: Reviewer response for version 1

**DOI:** 10.5256/f1000research.139788.r164257

**Published:** 2023-03-30

**Authors:** Airton Tetelbom Stein, Rita Mattiello

**Affiliations:** 1 Graduate Program of Health Science, Public Health Department, Federal University of Health Science of Porto Alegre, Porto Alegre, Brazil; 2 Grupo Hospitalar Conceição, Porto Alegre, Brazil; 3 Post-Graduate Program in Epidemiology, Universidade Federal do Rio Grande do Sul, Porto Alegre, State of Rio Grande do Sul, Brazil

This protocol proposes an approach to assessing the place of residence as a spatial determinant of health in cities where traffic congestion might impact health services accessibility. The study provides dynamic travel times presenting data in ways that help shape decisions and spur action by diverse stakeholders and sectors. 

 This is a very adequate protocol to discuss a valid equity assessment in accessibility to health system and social justice.

 In the Introduction section, it could have a broader view and suggest to include this article for rationale - Hosking J, Braubach M, Buss D, Khayesi M, Filho VP, de Sá TH. Towards a global framework for transport, health and health equity. Environ Int. 2022 Nov;169:107472. doi: 10.1016/j.envint.2022.107472. Epub 2022 Aug 17. PMID: 36116365.

 There is a need to include in the introduction an article discussing the PROGRESS-Plus strategy, such as: O'Neill J, Tabish H, Welch V, Petticrew M, Pottie K, Clarke M, Evans T, Pardo Pardo J, Waters E, White H, Tugwell P. Applying an equity lens to interventions: using PROGRESS ensures consideration of socially stratifying factors to illuminate inequities in health. J Clin Epidemiol. 2014 Jan;67(1):56-64. doi: 10.1016/j.jclinepi.2013.08.005. Epub 2013 Nov 1. PMID: 24189091.

 There is a need to discuss this report - " *Integrating health in urban and territorial planning: A sourcebook for urban leaders, health and planning professionals*" - https://unhabitat.org/integrating-health-in-urban-and-territorial-planning-a-sourcebook-for-urban-leaders-health-and, in which it describes the need to build habitable cities on a habitable planet: Processes to guide the development of human settlements – in this document referred to as “urban and territorial planning (UTP)”; and concern for human health, well-being and health equity at all levels – from local to global, and from human to planetary health. As there is a need for a closer relationships between public health and spatial planning.

 Population ageing is one of the main demographic phenomena in Latin America and the Caribbean and in the world. This topic should also be addressed in this protocol, as this population has several disabilities due to chronic illness and inequity is something to manage based on this demographic transition.

Is the study design appropriate for the research question?

Yes

Is the rationale for, and objectives of, the study clearly described?

Yes

Are sufficient details of the methods provided to allow replication by others?

Yes

Are the datasets clearly presented in a useable and accessible format?

Yes

Reviewer Expertise:

I am a family physician and epidemiologist. My main interest in research is health service research, primary health care and clinical guidelines.

We confirm that we have read this submission and believe that we have an appropriate level of expertise to confirm that it is of an acceptable scientific standard.

## References

[1] : Towards a global framework for transport, health and health equity. *Environ Int* .2022;169: 10.1016/j.envint.2022.107472 107472 10.1016/j.envint.2022.107472 36116365

[2] : Applying an equity lens to interventions: using PROGRESS ensures consideration of socially stratifying factors to illuminate inequities in health. *J Clin Epidemiol* .2014;67(1) : 10.1016/j.jclinepi.2013.08.005 56-64 10.1016/j.jclinepi.2013.08.005 24189091

[3] Integrating health in urban and territorial planning: A sourcebook. *Geneva: UN-HABITAT and World Health Organization* .2020; Reference source
